# The Evaluation of Orthotics in Reducing Hallux Valgus Angle in Patients with Hallux Valgus over a Twelve-Month Treatment

**DOI:** 10.3390/ijerph191912531

**Published:** 2022-10-01

**Authors:** Guoli Li, Jinsong Shen, Edward Smith, Chetna Patel

**Affiliations:** 1 School of Art and Design, Guangzhou Panyu Polytechnic, Guangzhou 511483, China; 2 Textile Engineering and Materials Research Group, School of Fashion and Textiles, De Montfort University, Leicester LE1 9BH, UK; 3 The Maths Learning Centre, De Montfort University, Leicester LE1 9BH, UK

**Keywords:** hallux valgus, hallux valgus angle, orthotics, evaluation, effectiveness, treatment

## Abstract

Background: Hallux valgus (HV) is one of the most common forefoot deformities among females, and its prevalence increases with age. This study aims to evaluate the effectiveness of three different types of orthotics on the reduction in hallux valgus angle (HVA) for patients with mild and moderate hallux valgus deformities. Methods: Twenty-six patients (42 feet) with mild or moderate HV participated in the treatment with three types of orthotics in the current study. Patients were divided into three groups depending on their HV severities and the consideration of different function of the orthotics. Orthotic Type 1 is a biomechanical style orthotic applied to moderated HV in Group 1. Orthotic Type 2 is a wrap style orthotic used on mild and moderate HV with two sub-groups: mild HV in Group 2A and moderate HV in Group 2B. Orthotic Type 3 is a gel style orthotic for mild HV. Patients were required to wear the orthotics for between 6 and 8 h per night over a period of 12 months. The HVA was measured every 3 weeks using a newly designed Measuring Block. A paired t-test was used to compare the differences between initial and final HVA at different stages of HVA treatment with orthotics. Results: After the 12-month treatment, for moderate HV patients treated with the Orthotic Type 1, their HVA reduced by 5.05° (95% CI 1.37, 8.73), (*p* < 0.05). For moderate HV patients treated with the Orthotic Type 2, their HVA reduced by 1.2° (95% CI −0.71, 3.11) (*p* > 0.05). For mild HV patients treated with the Orthotic Type 2, their HVA reduced by 2.44° (95% CI 1.39, 3.49) (*p* < 0.05). For mild HV patients treated with the Orthotic Type 3, their HVA reduced by 3.08° (95% CI −0.68, 6.83) (*p* > 0.05). Conclusions: Orthotic Type 1 showed a consistent significance in reduction in the HVA during the 12-month treatment, so it could be recommended for treating moderate HV. Orthotic Type 2 reduced the HVA, but it did not show a consistent significance in reduction in the HVA for mild and moderate HV. Orthotic Type 3 reduced the HVA, but it showed a volatile trend during 12 months without significant differences.

## 1. Introduction

Hallux valgus (HV) is one of the most common forefoot deformities among females, and its prevalence increases with age [1,2]. HV has been associated with pain in the big toe [3], poor foot function, difficulty in fitting footwear [4,5], and a health-related poor quality of life [6,7]. The exact aetiology of HV remains unclear [8]. The causes of HV are multifactorial and complex, which might consist of the effects from wearing improper footwear, abnormalities in foot anatomy and foot mechanics, limb inequality, occupational hazards, inflammatory joint diseases, and genetic and neuromuscular factors [9].

There are more than 200 different surgical techniques for the treatment of HV [10], but these techniques have not provided a comprehensive solution. Many surgical procedures have been described in the literature for the correction of the HVA, but there are many possible risks from HV surgery including infection, nerve injury, failure to relieve pain, and stiffness of the big toe joint. Patients could take a long time for their recovery back to normal activities after the costly surgery. Many studies have stressed that surgical treatments could lead to other complications [11,12] or recurrent HV. There is a high rate of dissatisfaction between 25% and 33% after surgery among HV patients [13].

Publications, reports, and studies have highlighted that non-surgical treatments should be used to correct the HVA for HV patients. Previous studies have tried to treat HV with orthotics such as a new foot-toe orthotic [14], an insole with toe separator [15], a night splint [16], and a custom-made foot orthotic [17]. However, the evidence for effectiveness of orthotics for patients with HV is very limited.

There are many different types of orthotics available on the market. However, there are inadequate studies and evidence to prove the effectiveness of existing orthotics on reducing the HVA from a long-term treatment. Therefore, there is still a lack of suggestion and recommendation of orthotics for HV patients from hospitals or clinical practice. There is little research evidence guiding podiatrists’ clinical decisions surrounding the non-surgical management of HV [18]. The reason might be because the effectiveness of orthotics has not been proven from users so that the current orthotics are difficult to be recommended. The aim of this study is to determine the effectiveness of currently available representative orthotics on reducing the HVA over a 12-month period of treatment for patients with mild to moderate HV.

## 2. Materials and Methods

### 2.1. Study Design

Thirty-two patients aged 18 and over with mild and moderate HV participated in this trial experiment. Six of the thirty-two patients dropped out due to personal reasons such as pregnancy, business commitments, or health. Therefore, 26 patients remained for the duration of the trial. These 26 patients had 42 feet (bilateral *n* = 16, unilateral *n* = 10) identified with HV based on the Manchester Scale method and, then, were tested.

In this trial, patients were divided into three groups depending on their HV severity as determined by the American Orthopaedic Foot and Ankle Society (AOFAS) angular measurements: mild HV (HVA < 20°) and moderate HV (HVA 20–40°). Group 1 (*n* = 12) consisted of moderate HV patients to be treated with a biomechanical style orthotic (Type 1). Group 2 (*n* = 23) consisted of both mild and moderate HV patients and was further divided into two sub-groups: mild HV patients in Group 2A (*n* = 15) and moderate HV patients in Group 2B (*n* = 8) to be treated with a wrap style orthotic (Type 2). Group 3 (*n* = 7) consisted of mild HV patients to be treated with a gel style orthotic (Type 3).

### 2.2. Criteria

The inclusion criteria were that the participants were aged 18 and over and suffered from mild or moderate HV deformity. The exclusion criteria for participants were previous bunion surgery or use of foot orthotics, rheumatoid disease, pregnancy, balance impairments, other aetiology arthritis, neurologic diseases, bone fractures in the last three years, cognitive disorders, diabetes, and inflamed or swollen feet. In addition, plantar orthoses were also excluded because pronation control could influence the development of the pathology.

### 2.3. Orthotics

**Figure 1:**
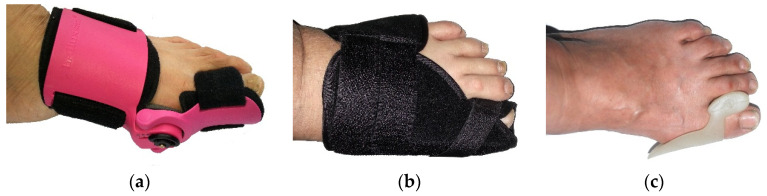
Three different types of orthotics used in the treatment trial. (**a**) Orthotic Type 1; (**b**) Orthotic Type 2; (**c**) Orthotic Type 3.

Three commercial and representative orthotics have been used in different groups of HV participants, as shown in Figure 1. The features of these orthotics are as follows:

Orthotic Type 1 is a biomechanical style. This orthotic provides a stronger support to the foot, and it can be adjusted to control the big toe position and to promote the alignment of the big toe. Wearing the biomechanical style orthotic allows the foot to be stretched out as much as possible and has more strength compared with the other two types of orthotics. The special function of this orthotic is that it has five different levels of adjustment, with the lowest being level 1 and the strongest being level 5.

Orthotic Type 2 is a wrap style, which includes an aluminum stay, Velcro fastening, and foam padding. This orthotics can adjust the big toe using the wrap to control the big toe in a correct alignment. There is a metal bar at the side of the bunion splint, which can hold the foot and help to improve the position of the big toe, stretching out the muscles, ligaments, and tendons. It is only suitable for wearing at night because it would be difficult to walk while wearing it due to the metal bar.

Orthotic Type 3 is a silicone gel style. This orthotic keeps the big toe and second toe apart, and it has advantages of being light, convenient, and easy to wear inside footwear.

### 2.4. Procedure

All patients were given oral and written study information and were required to give written consent to participate in the study based on an assigned ethical form. The ethics for this study was approved by the Faculty Research Ethics Committee of De Montfort University, Leicester, UK. Patients were divided into three groups depending on their HV severity. All patients were examined by an orthopaedic surgeon before treatment. In Group 1, twelve participants with moderate HV were provided with Orthotic Type 1. The patients were asked to adjust Orthotic Type 1 by increasing one level every two months from weakest level of adjustment up to the strongest level of 5. In Group 2, fifteen patients with moderate HV and eight participants with mild HV were provided with Orthotic Type 2. Orthotic Type 2 was used to adjust the wrap to control the big toe in a correct alignment at the level when patient can feel the pain but at an acceptable level of toleration. In Group 3, seven participants with mild HV were provided with Orthotic Type 3. All patients were required to wear the orthotics for between 6 and 8 h per night over a period of 12 months, and patients were advised to avoid wearing high-heel or pointed toe footwear during treatment. The HVA was measured at 3 weeks intervals during the 12 months periods by the same researcher following the same operating procedure.

### 2.5. Measuring Device

**Figure 2:**
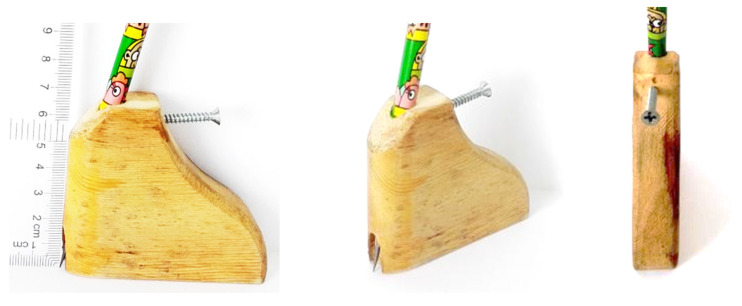
Design and characteristics of a newly designed measurement device.

A newly designed Measuring Block (Figure 2) was used for this current study. This device’s validity and reliability have been approved in a study by Li et al. [19]. The HVA measurement was taken by using this Measuring Block whilst the patient was in a non-weight-bearing sitting position.

### 2.6. Measuring Method for Determining HVA Using the Measurement Device

**Figure 3:**
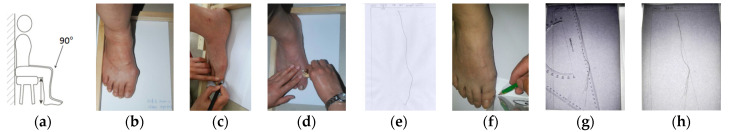
Standard Operating Procedure (**a**–**h**) of measuring the HVA [19].

The Measurement Block device was used to determine the HVA. To improve the reliability of HVA measurement, a standard measuring procedure was set up as follows:

(a) Participants sat barefoot, keeping a straight back on a chair. The chair was adjusted to ensure that the participant’s lower leg was vertical and at 90° relative to the upper leg. The participant’s feet were placed flat on the floor without bearing body weight (Figure 3a).
(b) A piece of A4 blank paper was placed underneath the foot of the participant. The new tool, called the right-angle wooden ruler, which was designed by the researcher and made by a carpenter, was used to keep the foot steady and to ensure that the heel and lateral foot shape were positioned at a 90° angle. The outside of the foot and the heel were placed as close as possible to the right-angle wooden ruler (Figure 3b).
(c) Three prominent points on the foot were located: the metatarsal phalange joint, the interphalangeal joint, and the navicular. These three points were marked using a cross on the foot to be measured (Figure 3c).
(d) The new measurement device was used to trace the outline of the foot from the heel to the end of the big toe (Figure 3d). The foot shape outline was traced on the A4 paper.
(e) A set square was used to record each of three prominent points (the medial border of the soft tissue of the big toe, the ball of the big toe, and the medial border of the heel) on the A4 paper (Figure 3e).
(f) A traced outline of the foot marked with the three prominent points was obtained on the A4 paper (Figure 3f).
(g) A ruler was used to draw two tangent lines by connecting the most prominent points between the metatarsal phalange joint and the navicular, as well as between the metatarsal phalange joint and the phalanges joint. Two tangent lines were extended to meet each other (Figure 3g).
(h) Finally, a protractor was used to record the HVA (Figure 3h).

### 2.7. Statistical Analysis

In the current study, data were analysed using the IBM SPSS Statistics Version 25 (IBM, Armonk, NY, USA). A paired *t*-test was used to compare differences of the HVA before and after treatments for the three orthotics. Descriptive Statistics Analysis was used to analyse minimum, maximum, mean, and Standard Deviation (SD) during different stages. A value of *p* < 0.05 is considered to indicate statistical significances.

## 3. Results

**Figure 4:**
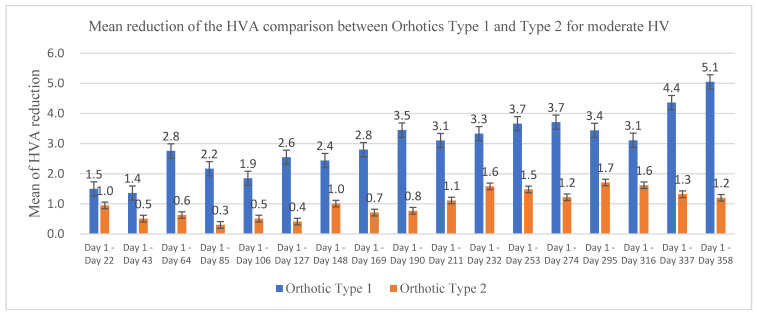
Mean of HVA reduction comparison between Orthotics Type 1 and Type 2 for moderate HV.

**Figure 5:**
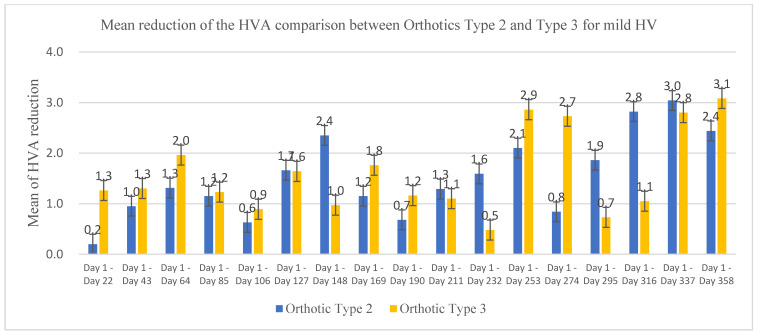
Mean of HVA reduction comparison between Orthotics Type 2 and Type 3 for mild HV.

The demographic characteristics of the patients including age, weight, height, and BMI are shown in Table 1. Twenty-six HV patients (42 feet) undertook treatment trials with orthotics over a period of 12 months. The HVA was measured every 3 weeks using the Measuring Block. A paired t-test was used to compare the differences between the initial and the final HVA at different stages of HVA treatment with orthotics. Figure 4 shows the mean of HVA reduction comparison between Orthotics Type 1 and Type 2 for moderate HV and Figure 5 shows the mean of HVA reduction comparison between Orthotics Type 2 and Type 3 for mild HV.

Table 2 shows the reduction in the patients’ mean HVA before and after treatments with three different types of orthotics. After the 12-month treatment, the Orthotic Type 1 reduced the HVA by 5.05° (95% CI 1.37, 8.73), (*p* < 0.05) for moderate HV. Importantly, there is a consistent reduction during 12 months of treatment (*p* < 0.05). Orthotic Type 2 reduced the HVA reduced by 2.44° (95% CI 1.39, 3.49) (*p* < 0.05) for mild HV and by 1.2° (95% CI −0.71, 3.11) (*p* > 0.05) for moderate HV. However, there were not consistent significances during the 12 month of treatment (*p* > 0.05). Orthotic Type 3 reduced the HVA by 3.08° (95% CI −0.68, 6.83) (*p* > 0.05) for mild HV.

## 4. Discussion

The objective of this research was to evaluate the effectiveness of three different types of orthotics on the reduction in the HVA for patients with mild and moderate HV over a 12-month period of treatment. There are many different types of orthotics available on the market. However, there are inadequate studies and evidence for long-term treatments to prove the effectiveness of existing orthotics on reducing the HVA. In this study, a comparison of the results between Orthotic Type 1 and Orthotic Type 2 for treating patients with moderate HV showed that the performance and effectiveness of Orthotic Type 1 on the reduction in the HVA were better than those of Orthotic Type 2. This might be because Orthotic Type 1 was more sophisticated, with five different levels of adjustment, which could be used to progressively increase the alignment angle to keep the big toe straight during the period of treatment matching the patients’ toleration level. In addition, Orthotic Type 1 was not easy to loosen when patients wore it at night because it had a cover around their feet. Therefore, Orthotic Type 1 is suggested to correct the HVA for moderate HV.

The mean HVA reduction between Orthotic Type 2 and Orthotic Type 3 for treating patients with mild HV was compared. When using Orthotic Type 2, the HVA was reduced by 2.44° (95% CI 1.39, 3.49) after the 12-month treatment. For mild HV patients, with a statistical significance of 95% confidence (*p* < 0.05), there was no consistent reduction in the HVA during the 12 months. Orthotic Type 2 seemed more reliable compared with Orthotic Type 3 for achieving a reduction in the HVA for mild HV patients, with 95% confidence. Although the Orthotic Type 3 also produced a big reduction in the HVA by 3.08° (95% CI −0.68, 6.83) (*p* > 0.05) after 12 months of treatment, there was no statistically significant difference at 95% confidence in the reduction in the HVA. Orthotic Type 3 seemed effective for the reduction in the HVA but without 95% confidence. This might be because some patients had an allergy to the gel materials and dropped out during the treatment trial, resulting in a reduction of the sample size. Further studies are suggested to use a larger sample size to evaluate the effectiveness of Orthotic Type 3 and the recurrence of the HVA in the longer term.

A previous trial study performed by Chadchavalpanichaya and Chueluecha [20] investigated the effectiveness of a commercial night-time HV strap (common night splint) on reducing the progression of the HVA. Forty-seven patients with moderate to severe HV (HVA 20–45°) participated in the study. Participants were divided into two groups: the study group (25 patients) and the control group (22 patients). The two groups of patients were very similar in terms of age, BMI, education levels, hours of work time, and shoe-wearing duration. Patients were asked to wear the night-time strap for 8 hours per night for a year. Their results showed no significant differences in the decrease in the HVA between the two groups (*p* > 0.05), although the night-time strap reduced the HVA by 1.16° (±2.60°), *p* > 0.05, after the 6-month treatment and by 0.8° (±3.70°), *p* > 0.05, after 12 months of treatment in the study group. Interestingly, in our current study, Orthotic Type 2 reduced the HVA by 0.77° (±2.56°), *p* > 0.05, after the 6-month treatment, and by 1.20° (±3.16°), *p* > 0.05, after the 12-month treatment in the moderate HV group. Compared with the Chadchavalpanichaya and Chueluecha study, the current study shows that the HVA was consistently reduced during the yearlong treatment but without 95% confidence. The reason may be that the overnight strap orthotic lacked strength to pull the big toe into a proper position for moderate HV. Another reason also can be that the orthotic may be loose during the night.

Mirzashahi et al. [21] also carried out a study to compare and evaluate the effectiveness of two splints: a newly designed slipper splint and a conventional splint commercially available on the market. Thirty patients were selected for study trials on these two splints. They were randomly divided into two groups for assessing their HVA every three months over a year. Patients wearing the prescribed splints for at least 8 hours a day on both feet while weight bearing were measured by anteroposterior radiography. Repeated tests and measurements were analysed. The results show that the HVA decreased marginally by 0.25° (±0.03°), *p* < 0.001 (on the left foot), and 0.28° (±0.14°), *p* < 0.001 (on the right foot), using the designed splints after a year treatment. The commercial orthotic also decreased the HVA by only 0.05° (±0.09°) (on the left foot) and by 0.08° (±0.14°) (on the right foot). Compared with our current study, Orthotic Type 2 reduced the HVA by 1.20° (±3.16°), *p* > 0.005, for moderate HV after 12 months of treatment and by 2.44° (±0.85°), *p* < 0.005, for mild HV after 12 months of treatment. Night splints or braces are mostly worn during rest or night time as the patient cannot walk while wearing the splints, as daily activities are affected. As these night splints or braces are bulky and not comfortable, patients might not wear them long enough during night, resulting in less effectiveness of treatment. Another reason might be that the night splint does not have sufficient strength to push the big toe to a normal position. Orthotic Type 2 was a wrap style, secured with a strap around the big toe. Different patients felt pressure or pain at different levels so some patients might wear the orthotic tightly but others might wrap the big toe too loosely. Due to these factors, it is difficult to compare the exact level of correction between patients during the trials.

In the current study, Orthotic Type 1 reduced the HVA by 3.45° (±393°), *p* < 0.05, after the 6-month treatment and by 5.05° (±5.15°), *p* < 0.05, after the 12-month treatment. Orthotic Type 1 showed more effectiveness than the night-time HV strap in reducing the HVA. Importantly, the reduction in the HVA by 5.05° had a 95% confidence level (95% CI 1.37, 8.73) (*p* < 0.05). Therefore, Orthotic Type 1 is more effective in reducing the HVA and is recommended for use as a conservative treatment for patients with moderate HV.

A previous study by Chadchavalpanichaya et al. [22] investigated the effect of using a custom-mold room-temperature vulcanizing silicon toe separator to decrease HVA. The HVA was measured at baseline, 6 months, and 12 months. After 6 months of using the orthotic, the HVA decreased by 2.2° ± 1.8°, *p* < 0.001. After 12 months of using the toe separators, the HVA of the study group decreased by 3.3° ± 2.4° from 32.0° ± 4.8° to 28.8° ± 5.8°. Compared with the Chadchavalpanichaya et al. study, we found that Orthotic Type 3 reduced the HVA by 1.16 ± 1.14° after 6 months of treatment, *p* > 0.05, and by 3.08° ± 2.36°, *p* > 0.05, after 12 months of treatment in our current study. There was no statistically significant difference at 95% confidence in the reduction in the HVA. Differently, Orthotic Type 3 was used to treat mild HV. Orthotic Type 3 is a gel style to keep the big toe and second toe apart, but there is a lack of strength in pulling the big toe outwards. This might cause a correction level inconsistency. In addition, some patients had an allergy to the gel materials and dropped out during the treatment trial, resulting in a reduction in the sample size.

The current study is subject to some limitations. The sample size for our treatment trials with three types of orthotics consisted of 26 patients (42 HV feet) in total. Spread over the 3 orthotics make sample size small. However, monitoring and measuring patients’ feet every 3 weeks over a year were time-consuming and costly, especially for patients who lived in different locations and towns. Furthermore, the orthotics were too expensive to be used for a larger sampling size. During the trial, some orthotics needed to be replaced because they broke or did not last long enough for the treatment period of 12 months. Therefore, the sample size was reduced in some of the treatment trials. That was due to the longer treatment trials being carried out over 12 months. In these trials, the patients were not sub-grouped based on their ages. The effectiveness of using the same orthotic might have been influenced by the ages of patients. This might be because young people seem to have more responses to the orthotic treatment. The effectiveness of the orthotics to reduce HV can also be largely influenced by adherence to the orthotics. Furthermore, patients’ walking activities might be another confounding factor affecting the effect of treatment during the trial. However, the amount of walking activity was not monitored for inclusion in the data analysis, which could be another limitation in this study.

HVA might have recurred or be aggravated if patients did not wear orthotics after 12 months. Long-term studies should be carried out to evaluate whether HV recurs or the HVA is back to a normal angle. Importantly, patients should also take into consideration avoiding wearing toe-pointed high heels that might aggravate HV. Further evaluation of orthotics with a larger sample size, different age groups, and repeat treatment sessions could be carried out in future work.

## 5. Conclusions

The current study evaluated the effectiveness of three different types of orthotics on the reduction in the HVA in patients with mild and moderate HV. For Orthotic Type 1 after 12 months of treatment for patients with moderate HV, there was a consistent reduction during the 12 months of treatment. Orthotic Type 1 was effective in reducing the HVA and can be recommended for patients with moderate HV. Orthotic Type 2 reduced the HVA for patients with mild HV and moderate HV after 12 months of treatment. However, there were no consistent significances on both these groups during the 12-month treatments. Therefore, Orthotic Type 2 was not recommended for moderate HV, and further evaluation of Orthotic Type 2 on mild HV with a larger sample size and repeat treatment could be carried out in future work. Orthotic Type 3 led to a big reduction in the HVA for mild HV after 12 months of treatment but without statistical significance. This might be because some patients had an allergy to the gel materials and dropped out during the treatment trial. Further study with a larger sample size might be needed prior to recommendations being made for Orthotic Type 3. The knowledge, information, and data from this evaluation on the effectiveness of three different type orthotics over 12 months of treatment can benefit hospitals, HV patients, orthotics manufacturers, and orthotic designers.

## Figures and Tables

**Table 1:** Demographic data of patients (n = 26).

Patients	N	Minimum	Maximum	Mean ± SD
Age (y)	26	20	72	53.27 ± 12.72
Height (cm)	26	140	173	157.92 ± 6.23
Weight (kg)	26	48	90	62.00 ± 9.47
BMI (kg/cm²)	26	18.7	31.3	24.84 ± 3.39

BMI: body mass index; SD: standard deviation.

**Table 2:** Comparison of three orthotics in correction of HVA over a 12-month treatment including 95% confidence interval of the difference and *p*-value.

	Moderate HV Treatment with Orthotic Type 1			Moderate HV Treatment with Orthotic Type 2			Mild HV Treatment with Orthotic Type 2			Mild HV Treatment with Orthotic Type 3		
	HVA (°) Reduction	95% (CI)	*p*- Value	HVA (°) Reduction	95% (CI) *	*p*- Value	HVA (°) Reduction	95% (CI) *	*p*- Value	HVA (°) Reduction	95% (CI) *	*p*- Value
Subjects	*n* = 12			*n* = 8			*n* = 15			*n* = 7		
Baseline (HVA)	26.94 ± 4.83			17.06 ± 1.72			27.83 ± 4.52			18.31 ± 1.41		
0–3 Months	2.17 ±3.31	0.07, 4.27	0.04 *	0.30 ±2.37	−1.01, 1.61	0.63	1.15 ±3.37	−1.67, 3.97	0.37	1.23 ±0.96	0.34, 2.12	0.02 *
0–6 Months	3.45 ±3.93	0.81, 6.08	0.02 *	0.77 ±2.56	−0.64, 2.19	0.26	0.68 ±2.64	−1.53, 2.88	0.49	1.16 ±1.14	−0.26, 2.58	0.09
0–9 Months	3.71 ±3.18	1.43, 5.99	0.01 *	1.22 ±3.31	−0.69, 3.14	0.19	0.84 ±2.33	−1.31, 2.99	0.38	2.73 ±2.11	−0.63, 6.08	0.08
0–12 Months	5.05 ±5.15	1.37, 8.73	0.01 *	1.20 ±3.16	−0.71, 3.11	0.20	2.44 ±0.85	1.39, 3.49	0.00*	3.08 ±2.36	−0.68, 6.83	0.08

* 95% confidence interval of the difference; * *p* < 0.05.
